# Exploration of Potential Roles of a New LOXL2 Splicing Variant Using Network Knowledge in Esophageal Squamous Cell Carcinoma

**DOI:** 10.1155/2014/431792

**Published:** 2014-08-31

**Authors:** Bing-Li Wu, Guo-Qing Lv, Hai-Ying Zou, Ze-Peng Du, Jian-Yi Wu, Pi-Xian Zhang, Li-Yan Xu, En-Min Li

**Affiliations:** ^1^Department of Biochemistry and Molecular Biology, Shantou University Medical College, Shantou, Guangdong 515041, China; ^2^Department of Pathology, Shantou Central Hospital, Affiliated Shantou Hospital of Sun Yat-sen University, Shantou, Guangdong 515041, China; ^3^Institute of Oncologic Pathology, Shantou University Medical College, Shantou, Guangdong 515041, China

## Abstract

LOXL2 (lysyl oxidase-like 2), an enzyme that catalyzes oxidative deamination of lysine residue, is upregulated in esophageal squamous cell carcinoma (ESCC). A LOXL2 splice variant LOXL2-e13 and its wild type were overexpressed in ESCC cells followed by microarray analyses. In this study, we explored the potential role and molecular mechanism of LOXL2-e13 based on known protein-protein interactions (PPIs), following microarray analysis of KYSE150 ESCC cells overexpressing a LOXL2 splice variant, denoted by LOXL2-e13, or its wild-type counterpart. The differentially expressed genes (DEGs) of LOXL2-WT and LOXL2-e13 were applied to generate individual PPI subnetworks in which hundreds of DEGs interacted with thousands of other proteins. These two DEG groups were annotated by Functional Annotation Chart analysis in the DAVID bioinformatics database and compared. These results found many specific annotations indicating the potential specific role or mechanism for LOXL2-e13. The DEGs of LOXL2-e13, comparing to its wild type, were prioritized by the Random Walk with Restart algorithm. Several tumor-related genes such as ERO1L, ITGA3, and MAPK8 were found closest to LOXL2-e13. These results provide helpful information for subsequent experimental identification of the specific biological roles and molecular mechanisms of LOXL2-e13. Our study also provides a work flow to identify potential roles of splice variants with large scale data.

## 1. Introduction

The lysyl oxidase (LOX) family, which is composed of five enzymes (LOX and LOXL1/2/3/4), catalyzes oxidative deamination of lysine residues in their protein substrates, generating highly reactive aldehyde residues that initiate inter- and intramolecular cross-linkages [1]. LOX family members are present in several human tissues, including the placenta, heart, lung, kidney, and pancreas [2–6], and are critical for multiple biological functions, such as growth, development, senescence, chemotaxis, and cell mobility [7]. LOXL2 has been emphasized in recent years because of its critical roles in carcinomas. Upregulation of LOXL2 has been detected in many tumor cell lines or clinical samples and also closely correlates with tumor invasion and metastasis [8–11]. LOXL2 protein distributes in either extracellularly or intracellularly [12]. Secreted LOXL2 is able to mediate extracellular matrix remodeling by upregulation of tissue inhibitor of metalloproteinase-1 (TIMP-1) and matrix metalloproteinase-9 (MMP-9) [13]. Intracellular LOXL2 is able to positively regulate the epithelial-mesenchymal transition (EMT) inducer Snail by enhancing Snail stability and functional activity and promoting EMT and tumor progression through downregulation of E-cadherin [14]. Moreover, LOXL2 modulates focal adhesions, tight junctions, and cell polarity complexes in basal breast carcinoma cells through activation of the FAK signaling pathway [15]. The mechanisms of intracellular LOXL2 action are not yet fully known. Recently, LOXL2 has been found to be associated with chromatin and reported to be involved in histone H3 deamination, a novel function that is dependent on the LOXL2 catalytic domain [16]. These analyses suggest the functions of LOXL2 in carcinoma are multifaceted and complicated. Therefore, delineation of LOXL2 function will provide a broad understanding of carcinogenesis.

In our previous study, LOXL2 was found to be overexpressed in esophageal squamous cell carcinoma (ESCC) cell lines and clinical samples and was significantly associated with lymph node metastasis [17]. Immunohistochemistry results showed the expression level of LOXL2 in ESCC is decreased in the nucleus but increased in the cytoplasm. Overall survival rates of ESCC patients with decreased nuclear expression or increased cytoplasmic expression of LOXL2 are significantly lower than those of the patients with the reverse expression pattern [17]. In a recent study, we identified a splice variant of LOXL2 lacking exon 13, denoted by LOXL2-e13, which is also expressed in ESCC cell lines and clinical samples [18]. To reveal the biological roles and molecular mechanisms of LOXL2 and its variants, we overexpressed wild-type LOXL2 (LOXL2-WT) and LOXL2-e13 in ESCC KYSE150 cell line and analyzed the mRNA profiles by the PrimeView Human Gene Expression Array (Affymetrix Corp., St Clara, CA, USA).

Hundreds and thousands of interactions between either extracellular or intracellular proteins compose a network. With recent advances in high-throughput technologies in protein-protein interactions (PPIs), network knowledge can give rise to understanding the biological function and dynamic behavior of cellular systems, generating new biological hypotheses and providing important clues for experimental verification [19–21]. In this study, two PPI subnetworks were generated by mapping DEGs of LOXL2-WT and LOXL2-e13 to the human PPI dataset. These DEGs were annotated by Functional Annotation Chart in the DAVID bioinformatics database. Annotations were compared to reveal the potentially specific roles or mechanisms of LOXL2-e13. This analysis can provide important clues for the future identification of specific roles of LOXL2-e13 from the viewpoint of system analysis.

## 2. Materials and Methods

### 2.1. Differentially Expressed Genes

The detailed manipulations of overexpression and microarray were prepared in another manuscript [18]. Briefly, LOXL2-WT and LOXL2-e13 were cloned into the pcDNA3.0 plasmid and overexpressed by transfection into ESCC KYSE150 cells (a generous gift from Professor Mingzhou Guo, Department of Gastroenterology and Hepatology, Chinese PLA General Hospital, Beijing, China). Transfection with an empty plasmid was used as a control. The mRNA expression profiles were analyzed by PrimeView Human Gene Expression Array (Affymetrix Corp., St Clara, CA, USA), and the raw data was normalized by the RMA algorithm. The mRNA expression profiles have been submitted to the GEO database (http://www.ncbi.nlm.nih.gov/geo) under accession number of GSE53645. The DEGs of LOXL2-WT versus empty plasmid (LOXL2-WT-DEGs) and LOXL2-e13 versus empty plasmid (LOXL2-e13-DEGs) were differentiated using a threshold of 1.5-fold change. Moreover, e13-WT-DEGs were also obtained by comparing the expression profile of LOXL2-e13 to that of LOXL2-WT using the same threshold.

### 2.2. Protein-Protein Interaction Subnetwork Generation

Human Protein Reference Database (HPRD) (http://www.hprd.org) is a database of human proteins, including protein-protein interactions, posttranslational modifications, enzyme-substrate relationships, and disease associations [22]. The Biological General Repository for Interaction Datasets (BioGRID) is a freely accessible database of physical and genetic interactions for various species, which is available at http://www.thebiogrid.org [23]. All PPI data were collected manually from the published literature. These two datasets are popular in PPI network research or other high-throughput data analyses because of their reliability and up-to-date release [24, 25]. The newest PPI data version of* Homo sapiens* species from these two databases was downloaded for our study, and we integrated them by removing the redundant interactions. There were 18595 unique proteins and 174552 interactions in this integrated PPI dataset, and we regarded it as the parent PPI network.

First, we used Cytoscape to map the LOXL2-WT-DEGs and LOXL2-e13-DEGs PPI datasets to the HPRD&BioGRID parent PPI network to generate the respective PPI subnetworks. Cytoscape (http://www.cytoscape.org) is a free software for visualizing, modeling, and analyzing molecular and genetic interaction networks and has been widely applied in the field of “omics” research (e.g., genomics, transcriptomics, proteomics, and metabolomics) [26]. In Cytoscape, the PPI network is presented by a graph in which each protein is represented by a node, and each interaction between two nodes is represented by an edge. To increase the reliability and limit the protein perturbation to a certain level, the DEG PPI subnetwork construction was limited to the first neighbor proteins of each DEG.

### 2.3. Network Topological Parameter Analysis

In order to gain insight into the organization and structure of the PPI network, NetworkAnalyzer (http://med.bioinf.mpi-inf.mpg.de/netanalyzer) was applied to analyze the network topological parameters [27]. In the study of networks, the degree of a node is the number of its connections to other nodes, and the degree distribution is the probability distribution of these degrees over the whole network. The node-degree distribution of PPI subnetworks follows a power law, one of the most important network topological characteristics, and was analyzed as we previously described [28]. Briefly, the edges in all networks were treated as undirected. Node-degree distribution *P*(*k*) is the number of nodes with a degree *k*. By fitting a line on datasets, the pattern of their dependencies can be visualized. NetworkAnalyzer considers only data points with positive coordinate values for fitting the line where the power law curve of the form *y* = *βxa* is transformed into a linear model ln⁡*y* = ln⁡*β* + *a*ln⁡*x* and the *R* ^2^ value (coefficient of determination) provides a measure of how well the data points fit to the curve. An *R* ^2^ value closer to 1 indicates a better fit.

### 2.4. Functional Annotation of Differentially Expressed Genes and Comparison

Functional Annotation Chart in the DAVID bioinformatics database (http://david.abcc.ncifcrf.gov/) is a tool to identify the enriched biological terms associated with a large gene list [29]. Compared to other similar enrichment analysis tools, Functional Annotation Chart has an extended annotation coverage, increasing from only GO to over 40 annotation categories, including protein sequence features, protein-protein interactions, protein functional domains, disease associations, pathways, homology, gene functional summaries, gene tissue expression, and literature. For example, INTERPRO annotation provides functional analysis of protein sequences by classifying them into families and predicting the presence of domains and important sites [30]. SMART annotation (Simple Modular Architecture Research Tool) allows the identification and annotation of genetically mobile domains and the analysis of domain architecture [31]. The LOXL2-e13-DEGs and LOXL2-WT-DEGs were submitted to the DAVID bioinformatics database and their Functional Annotation Chart results obtained. The terms from the Functional Annotation Chart results with *P* < 0.05 were visualized by the Enrichment Map plugin in Cytoscape, which is a network-based visualization method for gene-set enrichment results [32]. The two Functional Annotation Chart results of LOXL2-e13-DEGs and LOXL2-WT-DEGs were compared and displayed.

### 2.5. Prioritization Analyses of e13-WT-DEGs

We applied the Random Walk with Restart (RWR) algorithm to analyze the prioritization of e13-WT-DEGs when considering their relationships with LOXL2-e13. RWR is an algorithm for graph analysis, for example, a PPI network, in which, at every tick time, a random walker has a chance to get back to one or more start nodes, from any current node, with a fixed, common, and constant probability [33, 34]. For example, when node(A) is a start node, an RWR result with node(A) provides the stationary probabilities from node(A) to all nodes of the given graph. These probabilities can be considered to be the affinity or proximity from node(A) to individual nodes. RWR is formally defined as the following equation:

(1) $$\begin{array}{c} p^{t+1}=\left(1-r\right)Wp^{t}+rp^{0}, \end{array}$$

where *r* is the restart probability, *W* is the column-normalized adjacency matrix of the network graph, and *p* ^*t*^ is a vector of size equal to the number of nodes in the graph where the ith element holds the probability of being at node *i* at time step *t*. In this study, RWR was executed with our custom *R* program in the e13-WT-DEGs PPI subnetwork with LOXL2 set as the seed node.

## 3. Results

### 3.1. Protein-Protein Interaction Subnetwork of Differentially Expressed Genes

Using a 1.5-fold change as the threshold, we obtained 349 DEGs, including 217 upregulated genes and 132 downregulated genes, from comparing LOXL2-e13 versus empty plasmid, while there were 606 upregulated genes and 351 downregulated genes from comparison of LOXL2-WT versusempty plasmid. A single protein could not work alone to achieve so many biological effects but must cooperate with other proteins through their physical interactions. To find how many and what kinds of proteins are connected with the DEGs, two PPI subnetworks were constructed by mapping the LOXL2-WT-DEGs and LOXL2-e13-DEGs to the HPRD&BioGRID parent PPI network to generate their respective PPI subnetworks.

**Figure 1:**
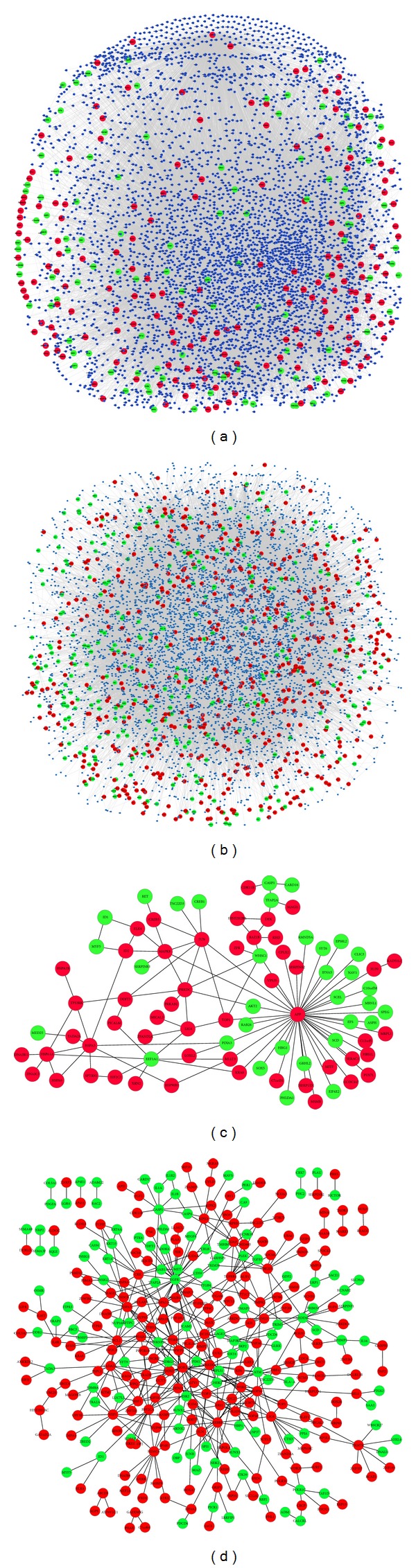
DEGs were mapped to the HPRD&BioGRID parent PPI network to generate PPI subnetworks. (a) PPI subnetworks of LOXL2-e13-DEGs. (b) PPI subnetworks of LOXL2-WT-DEGs. (c) Interactions between LOXL2-e13-DEGs. (d) Interactions between LOXL2-WT- DEGs. Nodes are labeled by different colors to indicate the expression trend of proteins. Green nodes represent proteins encoded by downregulated genes, while red nodes represent proteins encoded by upregulated genes. Interacting proteins without significantly different expression are represented as blue nodes.

The LOXL2-e13-DEGs PPI subnetwork contained 4768 nodes and 67769 edges, including 289 DEGs (Figure 1(a)). We also found, among the interactions between DEGs, a small protein cluster containing 84 nodes and 103 edges (Figure 1(b)). The LOXL2-WT-DEGs PPI subnetwork was composed of 6429 nodes and 97233 edges with 821 DEGs (Figure 1(c)). The interactions between LOXL2- and WT-DEGs were also found. To our surprise, there was a huge protein cluster containing 318 DEGs. These two PPI subnetworks indicated that overexpression of LOXL2-WT or LOXL2-e13 greatly perturbed the PPI network in ESCC cells due to DEGs interacting with hundreds and thousands of proteins to achieve the biological consequences of the LOX2 protein itself. To our great interest, a large number of interactions between DEGs were found, indicating these DEGs might form protein complexes in specific temporal or spatial patterns, and their upregulation or downregulation might favor their counterpart to conduct its biological functions.

### 3.2. Topological Parameters of PPI Subnetworks

**Figure 2:**
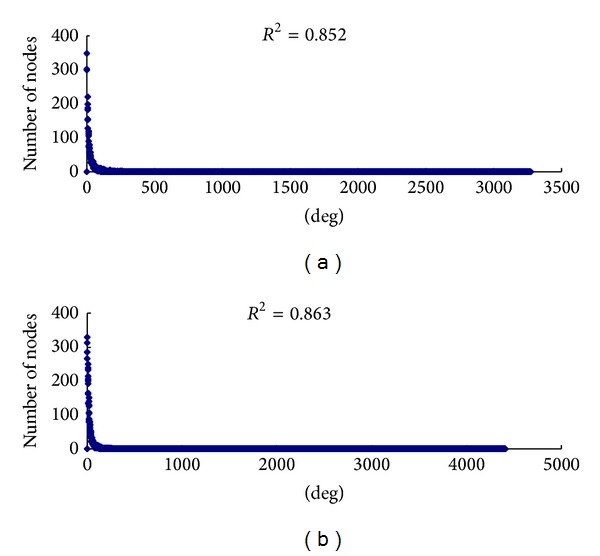
Power law fit of node-degree distribution for PPI subnetworks. The node degree (*k*) is represented on the *x*-axis and the number of nodes with *k* is represented on the *y*-axis. The graph displays a decreasing trend of degree distribution with an increase in the number of links, indicating scale-free topology. (a) Node-degree distribution of the LOXL2-e13-DEGs PPI subnetwork. (b) Node-degree distribution of LOXL2-WT-DEGs PPI subnetwork.

Network topological parameters are important characteristics for evaluating the networks. In this study, PPI subnetwork topological parameters were analyzed by NetworkAnalyzer. The distributions of node degree approximately followed power law distributions with *R* ^2^ = 0.863 and 0.852, respectively (Figure 2). Thus, these two PPI subnetworks were characterized to be scale-free, one of the most important parameters to recognize a true complex biological network [35]. The other topological parameters of these two subnetworks, such as clustering coefficient, network centralization, and network density, are shown in Table 1.

### 3.3. Annotations and Comparison of DEGs

To understand the function of these DEGs beyond the traditional gene listing and GO annotation, we submitted the LOXL2-e13-DEGs and LOXL2-WT-DEGs to Functional Annotation Chart analysis in the DAVID bioinformatics database. The Functional Annotation Chart results with *P* < 0.05 were visualized and displayed as a network by Enrichment Map.

**Figure 3:**
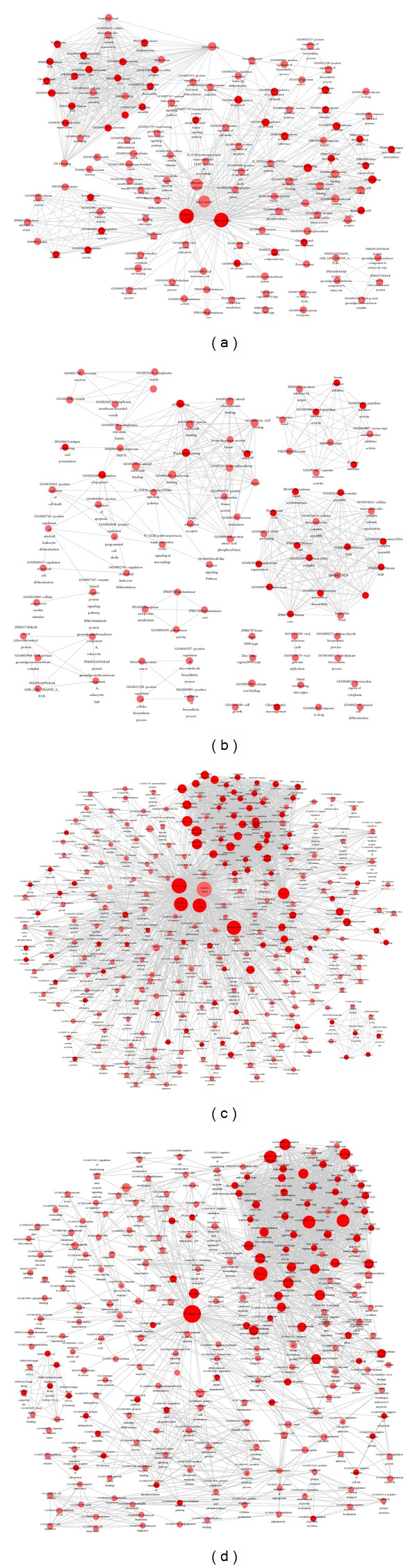
Visualization of Functional Annotation Chart analysis for DEGs and their comparison. (a) Visualization of Functional Annotation Chart analysis of LOXL2-e13-DEGs. (b) Visualization of Functional Annotation Chart analysis of LOXL2-WT-DEGs. (c) The unique Functional Annotation Chart of LOXL2-e13-DEGs. (d) The unique Functional Annotation Chart of LOXL2-WT-DEGs.

In Figure 3, the functional annotation terms were represented as nodes. The node with more significance was larger. Edge width was defined by the overlap coefficient between these categories (overlap coefficient cut-off was set as 0.6). The more genes shared by two nodes, the wider their edge width. For LOXL2-WT-DEGs, there were 166 terms from GO categories (e.g., biological process, cellular component, and molecular function) (Figure 3(a)). Except for these, another 96 terms were included from the following annotation categories: 13 INTERPRO, 3 SMART, 31 SP_PIR_KEYWORDS, 38 UP_SEQ_FEATURE, and 5 KEGG_PATHWAY. For the LOXL2-e13-DEGs, in addition to the 71 terms from GO categories, the Functional Annotation Chart results also contained another 51 terms from the following annotation categories: 12 INTERPRO, 2 SMART, 17 SP_PIR_KEYWORDS, 8 UP_SEQ_FEATURE, and 5 KEGG_PATHWAY (Figure 3(b)). These results provide more information than merely using GO enrichment alone, including protein sequence features, protein domains, and pathways.

To illustrate the specific characters or functions of LOXL2-e13 in comparison to wild type, we compared and displayed the differences of the two Functional Annotation Chart results. The number of unique annotation terms for LOXL2-e13 was 89, while that for LOXL2-WT was 229 (Figures 3(c) and 3(d)). From INTERPROT annotation, the protein sequences of LOXL2-e13-DEGs were mainly comprised of domains for “IPR007125:Histone core,” “IPR009072:Histone-fold,” and “IPR000558:Histone H2B.” LOXL2-e13-DEGs were only annotated by the terms for “IPR007872:Zinc finger, DPH-type.” To our surprise, LOXL2-WT-DEGs proteins showed several different zinc annotations, including “IPR007087:Zinc finger, C2H2-type,” “IPR015880:Zinc finger, C2H2-like,” and “IPR013087:Zinc finger, C2H2-type/integrase, DNA-binding” from INTERPROT; “GO:0008270~zinc ion binding” from GO “molecular function”; “zinc-finger,” “zinc finger region : C2H2-type (1–17), C2H2-type 19, and C2H2-type 21” from SP_PIR_KEYWORDS annotation. Other sequence feature annotations from PIR_SUPERFAMILY indicated LOXL2-e13-DEGs were characterized by “histone H2B,” “chaperone HSP70,” and “serpin,” while LOXL2-WT-DEGs were annotated by “zinc finger protein ZFP-36.” A specific KEGG pathway potentially correlated to carcinoma, “hsa04620:Toll-like receptor signaling pathway,” was found for the LOXL2-e13-DEGs network. As for LOXL2-WT-DEGs, four carcinoma-related KEGG pathways were found: “hsa04010:MAPK signaling pathway,” “hsa04110:Cell cycle,” “hsa04115:p53 signaling pathway,” and “hsa05200:Pathways in cancer.”

Both LOXL2-WT and LOXL2-e13 were involved in regulation of gene expression, but they might play different roles or at different levels. It showed LOXL2-e13-DEGs might affect chromosome remodeling, such as “GO:0006334~nucleosome assembly,” “GO:0031497~chromatin assembly,” “GO:0006333~chromatin assembly or disassembly,” “GO:0006323~DNA packaging,” “GO:0000786~nucleosome,” and “GO:0065004~protein-DNA complex assembly” from GO annotation. However, LOXL2-WT-DEGs might regulate expression mainly at a transcriptional level, as indicated by “GO:0045449~regulation of transcription,” “GO:0006350~transcription,” “GO:0006355~regulation of transcription, DNA-dependent,” “GO:0010629~negative regulation of gene expression,” “GO:0016604~nuclear body,” and “GO:0000775~chromosome, centromeric region.”

To our great interest, these two charts also provided clues revealing the different roles of LOXL2-WT and LOXL-e13 in malignancy. The GO “biological process” annotation showed LOXL2-WT-DEGs were involved in cell motility (GO:0048870), cell migration (GO:0016477), and regulation of cell adhesion (GO:0030155). Though LOXL-e13-DEG was not annotated by such terms related to the malignant degree of tumors, it could not be excluded that LOXL2-e13 also plays important roles in carcinoma development. Significantly, LOXL2-e13-DEGs could regulate the activities of several enzymes or modify proteins, including enzyme inhibitor activity (GO:0004857), Rab-protein geranylgeranyltransferase complex (GO:0005968), endopeptidase inhibitor activity (GO:0004866), glutaminase activity (GO:0004359), peptidase inhibitor activity (GO:0030414), serine-type endopeptidase inhibitor activity (GO:0004867), protein serine/threonine kinase activity (GO:0004674), and GO:0016310~phosphorylation.

### 3.4. Prioritization of e13-WT-DEGs

**Figure 4:**
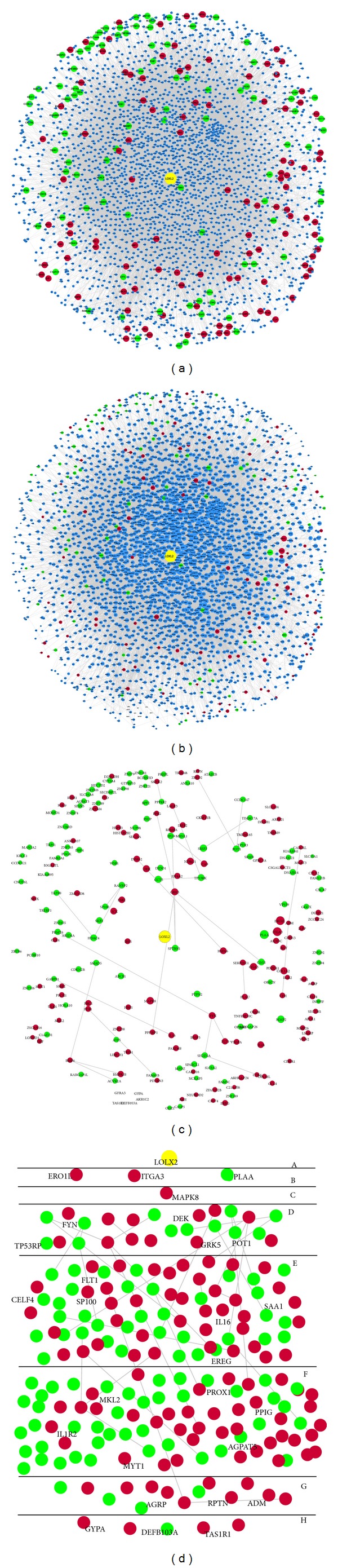
Prioritization analyses of e13-WT-DEGs based on the PPI subnetwork. (a) PPI subnetwork of e13-WT-DEGs. (b) Random Walk with Restart algorithm was used to score all proteins in the PPI network with LOXL2 set as the seed node. The size of each node in the PPI subnetwork was designed as a gradient based on the scores. (c) The e13-WT-DEGs were extracted from (b) to illustrate their sizes. (d) The DEGs were rearranged according to their closeness to LOXL2-e13 protein.

To provide a deep view of the different influence on ESCC cells between LOXL2-e13 and its wild type, we selected the DEGs between LOXL2-e13 and its wild-type counterpart for future analyses. We obtained 275 e13-WT-DEGs, including 118 downregulated genes and 157 upregulated genes. To obtain a full view of the influence of the e13-WT-DEGs, its PPI subnetwork was also constructed and contained 2741 nodes and 43507 edges, including 100 upregulated genes and 54 downregulated genes (Figure 4(a)). These results indicated that overexpression of LOXL2-e13 had a widely different impact on the mRNA expression profile compared to wild-type LOXL2, providing important clues to reveal the specific functions and potential molecular mechanisms of LOXL2-e13.

We applied the RWR algorithm to identify how the e13-WT-DEGs would be ranked by their closeness in the context of LOXL2-e13 overexpression. The RWR algorithm gave each protein member in the e13-WT-DEGs PPI subnetwork a probability score, which ranged from −2.56 to −8.74 (the more negative the score, the less significant the protein) (Figure 4(b)). Subsequently, only the nodes of e13-WT-DEGs were extracted from the modified PPI subnetwork for a better illustration (Figure 4(c)). Since some of the e13-WT-DEGs scored closely, we rearranged e13-WT-DEGs after their scores were log10-transformed and classified by the score ranges. For example, only the seed node LOXL2 was classed as the A layer; DEGs with a log-transformed score of −2.0~−2.99 were classified as the B layer, and DEGs with a log-transformed score of −3.0~−3.99 were classified as the C layer (Figure 4(d)). We found upregulated ERO1L and ITGA3 and downregulated PLAA ranked in the first class close to LOXL2-e13-DEGs and upregulated MAPK8 ranked in the second class. These results provided the priorities of e13-WT-DEGs when considering their relationships with LOXL2-e13, which could be applied as candidate genes for subsequent experimental identification.

## 4. Discussion

Esophageal squamous cell carcinoma (ESCC), the major histopathologic form of esophageal cancer, is one of the most prevalent cancers in Asia and is the fourth leading cause of cancer death in China [36, 37]. The functions of a splice variant might be different and vary compared to wild type [38]. Much evidence has indicated that splicing abnormalities are a hallmark of cancer [39–41]. The potential roles for splice variants in cancer might involve cell migration, cell growth, hormone responsiveness, apoptosis, and response to chemotherapy [42]. For example, two splice variants of K-Ras, K-Ras 4A, and 4B, which arise from two alternative versions of exon 4, have been found [43]. These two variants show antagonistic biological effects; K-Ras 4A exerts proapoptotic effects, while K-Ras 4B is an antiapoptotic protein. Both variants are coexpressed in many tissues, but their ratio is altered in sporadic colorectal cancer, favoring the antiapoptotic 4B isoform [44]. This indicates that the expression of tumour-specific splice variants significantly affects many cellular events, critical for cancer biology, which are still far from being illustrated.

Inspired by this evidence, we overexpressed LOXL2-e13 and determined the mRNA expression profile, in comparison with wild-type LOXL2 overexpression, to explore its specific biological roles. PPI subnetworks were generated by mapping the DEGs to a public PPI dataset to gain a full view of their influence. We show that LOXL2-WT-DEGs and LOXL2-e13-DEGs interact with thousands of other proteins, which suggests LOXL2-WT and LOXL2-e13 can greatly impact cellular activity through the cascades of interactions of the DEGs.

To better illustrate their potential molecular functions, Functional Annotation Chart analysis was applied to classify the DEGs based on related multiple gene function annotations, which allows investigators to analyze genes from many different biological aspects in a single space. Our results indicate that there are distinguishing differences between LOXL2-e13 and LOXL2 in regard to GO enrichment, protein sequence features, protein domains, and pathways. LOXL2-e13-DEGs contain a list of upregulated histone proteins, including HIST1H2AC, HIST1H2BC, HIST1H2BD, HIST2H2BE, HIST1H2BJ, HIST1H2AK, HIST2H4A, and HIST2H4B, which mediate DNA organization and play a dominant role in regulating eukaryotic transcription. On the other hand, there are as many as 78 proteins, containing zinc finger, which can interact with LOXL2-WT-DEGs, such as ZNF19, ZNF292, KLF11, CREB5, SNAI2, and SP2. It has been suggested that zinc-finger-containing proteins function in gene transcription, translation, mRNA trafficking, cytoskeleton organization, epithelial development, cell adhesion, protein folding, chromatin remodeling, and zinc sensing [45].

The other evidence for regulation of transcription by both DEG networks comes from the GO annotations, which show that LOXL2-e13-DEGs might affect chromosome remodeling, based on annotations “nucleosome assembly,” “DNA packaging,” and “protein-DNA complex assembly.” LOXL2-WT-DEGs might regulate expression mainly at the transcriptional level, such as “regulation of transcription” and “regulation of transcription, DNA-dependent.” These results suggest that both LOXL2-WT and LOXL2-e13 are involved in gene expression regulation at different levels. This could be one of the specific molecular mechanisms for LOXL-e13 compared to wild type.

The other distinguishing property for LOXL2-e13 is that its DEGs involve enzyme activities or modifications, such as “Rab-protein geranylgeranyltransferase complex,” “serine-type endopeptidase inhibitor activity,” and “peptidase inhibitor activity.” The term “serine-type endopeptidase inhibitor activity” contained 7 genes, including APP, SERPINB2, SERPINB1, SLPI, SERPINB3, SERPINB13, and OVOS2. SERPINB1 (serine protease inhibitor, clade B, member 1) has been shown to be downregulated in hepatocellular carcinoma and is correlatively related with cancer metastasis and intravasation [46]. Interference of SERPINB1 promotes migration and invasion of HCC cells, with an apparent increase in the level of active matrix metalloproteinase-2 (MMP2) [46]. It is interesting to note that SERPINB1 is also decreased 1.58-fold upon LOXL2-e13 overexpression. The “peptidase inhibitor activity” annotation contains 10 genes, including APP, CARD16, SERPINB2, BIRC6, SERPINB1, SLPI, SERPINB3, HSPA5, SERPINB13, and OVOS2. BIRC6 is upregulated 1.56-fold in the LOXL2-e13 expression profile. BIRC6 is a member of the inhibitors of apoptosis protein (IAP) family, which is thought to protect a variety of cancer cells from apoptosis [47]. BIRC6 has been found to be overexpressed in various carcinomas, such as prostate cancer, melanoma, and non-small-cell lung cancer [47–49]. The relationship between LOXL2 and metabolism was not found. We assumed that LOXL2-e13 might influence metabolism in ESCC cells and that this might be a specific biological role or molecular mechanism for LOXL2-e13.

It was important to identify which e13-WT-DEG responds to explain differing functions for LOXL2-e13. We prioritized the e13-WT-DEGs by application of the RWR algorithm based on current knowledge of PPI networks. For example, ERO1L, ITGA3, PLAA, and MAPK8 were DEGs that ranked the closest to LOXL2-e13. It was reported that the upregulation of ERO1L (ERO1-like (*S. cerevisiae*)) is specifically induced in hypoxic microenvironments coinciding with that of upregulated VEGF expression in human tumors, such as in hepatocellular carcinoma and glioblastoma [50]. In support of this, we find VEGF is upregulated 1.68-fold in the e13-WT-DEGs. ITGA3 (integrin, alpha 3) plays an important role in tumors. Loss of ITGA3 prevents skin tumor formation by promoting epidermal turnover and depletion of slow-cycling cells [51]. ITGA3 also serves as a biomarker for various conditions, such as a biomarker for estimation of the risks of locoregional and hematogenous dissemination of oral squamous cell carcinoma, a marker for NMuMG cells undergoing epithelial-mesenchymal transition and for cancer cells with aggressive phenotypes, and a diagnostic marker for the clinical outcome of tongue squamous cell carcinoma [52, 53]. MAPK8, also named JNK, is involved in a wide variety of cellular processes, such as proliferation, differentiation, transcription regulation, and development in both normal tissues and tumors [54]. So it is expected that LOXL2-e13 causes broad changes in mRNA expression profile, including some critical tumor-related genes, enabling LOXL2-e13 to play new and specific roles in ESCC compared to its wild-type counterpart.

## 5. Conclusions

In summary, we provide evidence, through generation, annotation, and comparison of PPI networks, to indicate that LOXL2-e13 might play different roles compared to wild-type LOXL2. These results provide helpful information in the experimental identification of its biological roles and explanation of its molecular mechanisms. With the development of high-throughput techniques for protein-protein interactions, the public PPI network database and its use in subsequent analyses will become more acute and reliable. Our analyses also provide a work flow to test the different roles of a splicing variant with large scale data.

## Figures and Tables

**Table 1:** Network parameters of the LOXL2-e13-DEGs and LOXL2-WT-DEGs PPI subnetwork.

PPI subnetwork	*y* = *βx* ^*a*^	*R* ^2^	Correlation	Clustering coefficient	Network centralization	Network density
LOXL2-e13	*y* = 2012.2*x* ^−1.319^	0.852	0.754	0.261	0.680	0.006
LOXL2-WT	*y* = 3763.3*x* ^−1.411^	0.863	0.618	0.242	0.681	0.005
